# CeL-ID: cell line identification using RNA-seq data

**DOI:** 10.1186/s12864-018-5371-9

**Published:** 2019-02-04

**Authors:** Tabrez A. Mohammad, Yun S. Tsai, Safwa Ameer, Hung-I Harry Chen, Yu-Chiao Chiu, Yidong Chen

**Affiliations:** 1 Greehey Children’s Cancer Research Institute, University of Texas Health Science Center at San Antonio, San Antonio, TX USA; 2 Department of Epidemiology and Biostatistics, University of Texas Health Science Center at San Antonio, San Antonio, TX USA

**Keywords:** Cell line authentication, Cell line identification, CeL-ID, RNA-Seq variant profiles, Mutation, SNP/Indel

## Abstract

**Background:**

Cell lines form the cornerstone of cell-based experimentation studies into understanding the underlying mechanisms of normal and disease biology including cancer. However, it is commonly acknowledged that contamination of cell lines is a prevalent problem affecting biomedical science and available methods for cell line authentication suffer from limited access as well as being too daunting and time-consuming for many researchers. Therefore, a new and cost effective approach for authentication and quality control of cell lines is needed.

**Results:**

We have developed a new RNA-seq based approach named CeL-ID for cell line authentication. CeL-ID uses RNA-seq data to identify variants and compare with variant profiles of other cell lines. RNA-seq data for 934 CCLE cell lines downloaded from NCI GDC were used to generate cell line specific variant profiles and pair-wise correlations were calculated using frequencies and depth of coverage values of all the variants. Comparative analysis of variant profiles revealed that variant profiles differ significantly from cell line to cell line whereas identical, synonymous and derivative cell lines share high variant identity and are highly correlated (*ρ* > 0.9). Our benchmarking studies revealed that CeL-ID method can identify a cell line with high accuracy and can be a valuable tool of cell line authentication in biomedical science. Finally, CeL-ID estimates the possible cross contamination using linear mixture model if no perfect match was detected.

**Conclusions:**

In this study, we show the utility of an RNA-seq based approach for cell line authentication. Our comparative analysis of variant profiles derived from RNA-seq data revealed that variant profiles of each cell line are distinct and overall share low variant identity with other cell lines whereas identical or synonymous cell lines show significantly high variant identity and hence variant profiles can be used as a discriminatory/identifying feature in cell authentication model.

**Electronic supplementary material:**

The online version of this article (10.1186/s12864-018-5371-9) contains supplementary material, which is available to authorized users.

## Background

Cell lines are an indispensable component of biomedical research and serve as excellent in vitro model systems in disease biology research including cancer. Cell lines are usually named by the researcher who developed them and till recently were lacking a standard nomenclature protocol [1–3]. This had led to cell line misidentification and poor annotation. In addition, cell lines also suffer from cross-contamination from other sources including other cell lines [1, 4]. All these factors affect overall scientific reproducibility. Common contaminants include Mycoplasma and other human cell lines including HeLa [5–8]. Cell line contamination is regarded as one of the most prevalent problems in biological research [1–5, 7] and the ongoing publication of irreproducible research is estimated to cost ~ 28 billion dollars each year in the USA alone [9]. Though cross contamination of cell lines have been acknowledged for almost 50 years [1–4, 9], very few researchers check for contaminations probably because of lack of access to cell authentication methods. Recently, however, the awareness towards the importance of authentication of cell lines has increased, and also NIH and various journals now require researchers to authenticate cell lines [1, 10]. It has been reported that approximately 15 to 20% of the cells currently in use have been misidentified [3, 11]. This includes many from the large datasets stored in public repositories [11].

Profiling of short tandem repeats (STRs) across several loci is the most common and standard test for cell line authentication as recommended by the Standards Development Organization Workgroup ASN-0002 of American Type Culture Collection (ATCC) [1, 2, 9–11]. However, unstable genetic nature of cancer cell lines such as microsatellite instability, loss of heterozygosity and aneuploidy in cancer cell lines, makes STRs based validation problematic [1–3]. Recent studies have also explored using more stable single nucleotide variant genotyping for cell line authentication either in combination with STR profiles or alone [1, 9, 11]. It has been shown that carefully selected panel of SNPs confers a power of re-identification at least similar to that provided by STRs [1, 9, 11–15]. Although many SNP based methods have been developed and are being used for cancer cell line authentication, these methods still suffer from lack of rapid access and not being cost effective.

With the advent and success of sequencing technologies, more and more researchers are using RNA sequencing to profile large amounts of transcript data to gain new biological insights. Moreover, RNA-seq data is also being used to identify single nucleotide variants in expressed transcripts [16]. It may be noted here that variants from RNA-seq cover around 40% of those identified from whole exome sequencing (WES) and up to 81% within exonic regions [17]. In a recent report, authors successfully re-identified seven colorectal cell lines by comparing their SNV profiles obtained from RNA-seq data to the mutational profile of these cell lines in COSMIC database [11, 18].

In this study, we present a RNA-seq based approach for Cell Line Identification (CeL-ID). We identify variants in each cell lines using RNA-seq data followed by pairwise variant profile comparison between cell lines using frequencies and depth of coverage (DP) values. Comparative analysis of variants revealed that variant profiles are unique to each cell line. Our benchmarking studies revealed that CeL-ID method can identify a cell line with high accuracy and can be a valuable tool for cell line authentication in biomedical research. In addition, using linear model regression technique, the approach can also reliably identify possible contaminator if requested. We choose to explore the utility of RNA-seq data in cell line authentication because it is the most commonly used technique among the seq-based methods and also relatively inexpensive, and we also demonstrated the minimum sequence reads requirement for each RNA-seq to maintain the authentication accuracy using a series of subsampling BAM files at 1million up to 50 million reads. With the popularity and accessibility of RNA-seq technology, a significant number of studies anyway involve the use of data from RNA-seq and hence the same can also be used to check the authenticity of the cell line.

## Methods

### CCLE dataset

**Fig. 1:**
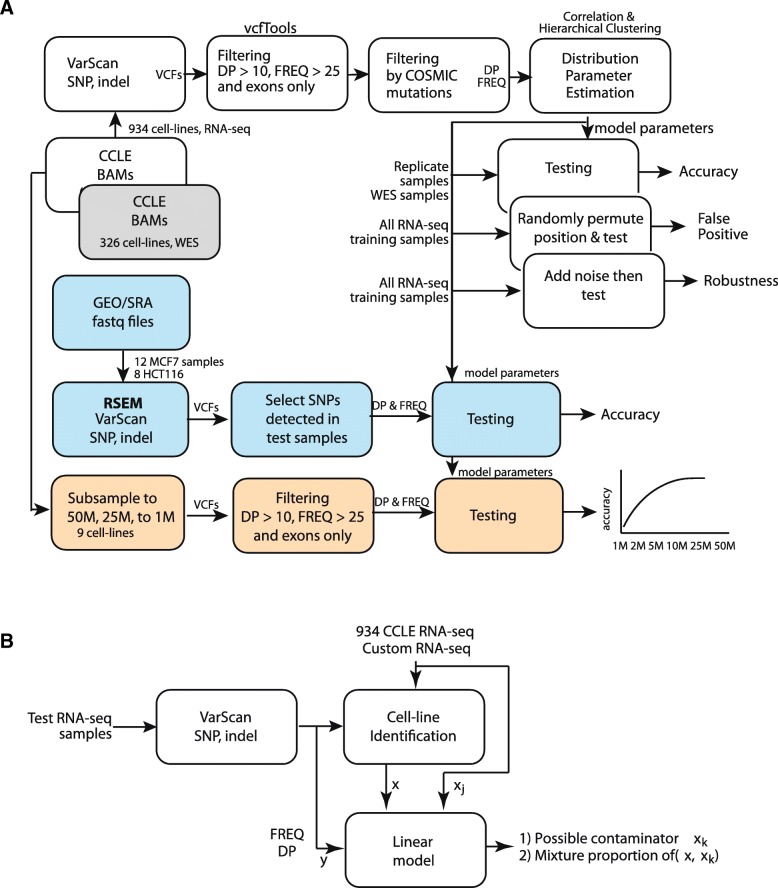
Schematic overview of CeL-ID method. **a** Shown are, in brief, the different steps involved in CeL-ID including evaluation of robustness of the model, testing on an independent dataset (light blue) and effect of subsampling on accuracy (light brown). **b** Flowchart of the contamination estimation model

The Cancer Cell Line Encyclopedia (CCLE) is a collaborative project focused on detailed genomic and pharmacologic characterization of a large panel of human cancer cell lines in order to link genomic patterns with distinct pharmacologic vulnerabilities and to translate cell line integrative genomics into clinic [19, 20]. Genomic data for around 1000 cell lines are available for public access and use. To be precise, National Cancer Institute (NCI) Genomic Data Commons (GDC) legacy archive hosts RNA sequencing data for 935 cell lines, whole exome sequencing (WES) data for 326 cell lines and whole genome sequencing (WGS) data for 12 cell lines (https://portal.gdc.cancer.gov/). The names of cell lines are used as is listed in NCI GDC archive and are listed in Additional file 1. We were able to download the RNA-seq bam files for all cell lines except one cell line named ‘G27228.A101D.1’ and whole exome sequencing bam files for all 326 cell lines. These bam files were processed using our in-house pipeline for variant calling. Variant calling process included removal of duplicate reads (samtools [21] and picard [https://broadinstitute.github.io/picard]), followed by local re-alignment and re-calibration of base quality scores (GATK [22]), and finally variant calling using VarScan [23] which includes both SNP and Indels. Downstream filtering (region-based to only include exome regions, sufficient coverage, and detectable allele frequency) and all other analyses were done using in-house Perl and MATLAB scripts. No filtering based on mutation types (specific to missense, nonsense or frameshift indels) or allele types (such as bi-allelic) were applied to CCLE samples. An illustrative depiction of the overall pipeline is shown in Fig. 1a. CCLE gene expression data were collected from (https://portals.broadinstitute.org/ccle/data) and it contains RPKM values for all the genes in 1019 cell lines, covering all 935 CCLE RNA-seq set.

### Independent RNA-seq datasets

We also used two publicly available RNA-seq datasets from GEO as independent test sets. First one is comprised of 12 MCF7 cell lines (GSE86316) whereas the second one has data for eight HCT116 cell lines (GSE101966) [24, 25]. These were generated to profile mRNA expression levels in MCF7 cells after silencing or chemical inhibition of *MEN1* [24] and in HCT116 cells after loss of *ARID1A* and *ARID1B* [25], respectively. We downloaded the fastq files for all these samples; aligned using RSEM [26] to align all reads to UCSC hg19 transcriptome, followed by variant calling using pipeline described earlier (Fig. 1a). We purposefully used a different aligner, RSEM [26], here to check the effect of different read aligners.

### Correlation and hierarchical clustering

To assess the confirmation of two cell-lines to be either identical or highly similar in terms of their sequence variation profiles genome-wide or their expression levels, we choose to use Pearson Correlation to evaluate altered allele frequencies (FREQ) across two cell-lines or expression levels, facilitated by the number of non-zero FREQ shared between two cell-lines with at least 10 fold coverage in both cell lines. We choose FREQ, instead of direct counting of altered allele depth (AD), because that majority of altered allele fractions does not change with the expression level, and allele-specific expression may appear in cell lines with certain treatments but hopefully it will be a small proportion over a typically massive number of SNPs under consideration. To be specific, for any two cell lines 〈 *i*, *j* 〉, the variants to be tested are

1 $$ V\in \left\{{V}_k,\kern0.5em where\kern0.5em {d}_{i,k}\ge \kern0.5em 10\kern0.5em \&\kern0.5em {d}_{j,k}\ge 10\kern0.5em \&\kern0.5em \left({f}_{i,k}>10\%\left|{f}_{j,k}>10\%\right.\right)\right\} $$

where *d*_*i,k*_ and *f*_*i,k*_ are the depth of coverage (DP) and altered allele frequency at genomic location *k* of *i*^*th*^ cell line, respectively. Note that we require variant has to exist in at least one cell line with 10 fold coverage. If a gene does not express, all mutations within this gene will not be considered unless its partner cell-line expresses this gene at a sufficient level. Therefore, the expression difference is already embedded in Pearson correlation, ${\rho}_{ij}={\sigma}_{ij}^2/{\sigma}_i{\sigma}_j$, where covariance and standard deviations will be evaluated over all variants in *V*. Similarly, correlations over gene expression levels between two cell lines are evaluated also by Pearson correlation coefficient, with requirement that genes with expression level > 0.1 (RPKM level) in at least one cell line. Hierarchical clustering was performed using MATLAB, using Pearson correlation of FREQ as the distance measure (over SNPs determined by Eq. 1), and with average linkage method.

To determine the significance of a detected correlation coefficient for a given cell line, we generated all pair-wise correlations for 934 RNA samples, and its distribution follows normal distribution *N*(*μ*, *σ*). Similar distribution is also observed in pair-wise correlation from WES samples. To estimate distribution parameters, we removed correlation coefficients less than 0 (unlikely) and greater than 0.8 (most likely due to replicate and derivative cell lines in CCLE collection), therefore it forms a truncated normal density function within an interval (*a*, *b*), as follows,

2 $$ f\left(x;\kern0.5em \mu, \sigma, \alpha, b\right)=\frac{\phi \left(\frac{x-\mu }{\sigma}\right)/\sigma }{\left(\Phi \left(\frac{b-\mu }{\sigma}\right)-\Phi \left(\frac{a-\mu }{\sigma}\right)\right)} $$

where we fixed cut-off *a* = 0, and *b* = 0.8. *ϕ* and *Φ* are standard normal density and distribution functions, respectively. We chose *b* = 0.8 as a cut-off threshold since pairs with correlation > 0.8 are derived from same parental lines or with some other biological relevance (see subsection *Cell line authentication using variant comparisons* in Results Section). Maximum-likelihood estimate (using MATLAB mle() function) was employed in this study, and distribution parameters from distribution (scaled to match the histogram setting) for CCLE collection were estimated. For any given correlation coefficient *ρ*_*i*_ for the test sample against *i*^th^ sample in CCLE, its *p* = *P*(*ρ* ≥ *ρ*_*ij*_) = 1 − *F*(*ρ*_*ij*_; *μ*, *σ*, *a*, *b*), where *F* is the cumulative distribution function of Eq. 2, we consider they are possibly related if *p* < 0.001, and they are most likely derived from same cell origin if *p* < 10^− 4^. Multiple samples are identified as matching cells, we can revise Eq. 1 to exclude all variants that shared from these matching cells, and then repeat the process.

For gene expression level, the distribution of pair-wise correlation coefficient is more skewed towards 1.0; therefore, it is difficult to separate matching cells from mismatch cells (data not shown).

### Contamination estimation using linear mixture model

In addition to authenticate cells, one may also want to know whether or not the processed cells are contaminated by other cells, possibly from CCLE or additional cell lines collected in the lab, along with RNA-seq data. Assuming the test sample is a mixture of cell lines *x*_1_ and *x*_2_, with unknown proportion *q*_1_ and *q*_2_, and we denoted the mixture cell as *y*, or,

3 $$ y\sim {q}_1{x}_1+{q}_2{x}_2+e $$

where *y*, *x*_1_, *x*_2_ are vectors of FREQs from selected variant sites of test mixture sample and CCLE cell lines. Eq. 3 can be re-formatted into matrix **Y** = **qX**, where **q** = [*q*_1_, *q*_2_, …], if more than two cell mixture is hypothesized. To demonstrate the proof-of-concept, our current implementation takes top 200 sites, each direction that has most difference in FREQ comparing two samples (total of 400 SNPs). To further simplify the procedure, we also use our CeL-ID to identify the dominant cell, say *x*_1_ first. Following the similar studies for de-convoluting cell type proportions [27, 28], we then test all 934 cell lines within CCLE collection, as *x*_2_, using robust linear model regression method (implemented in MATLAB fitlm() function) to estimate *q*_1_ and *q*_2_, provided *q*_1_ + *q*_2_ ≤ 1. Slightly different to typical cell-type deconvolution methods, after determining the first contaminator, we can iteratively add other candidates from the entire CCLE collection and perform linear regression, and terminate the process until *q* value becomes negative or regression fails (Fig. 1b).

We designed a simulation procedure to evaluate the effectiveness of the robust linear model *y*, by the following method,

4a $$ z={x}_1\bullet N\left({q}_1,{\sigma}_{q_1}\right)+{x}_2\bullet N\left({q}_2,{\sigma}_{q_2}\right) $$

4b $$ y=\left\{\begin{array}{cc}0& N\left(z,{\sigma}_f\right)<0\\ {}N\left(z,{\sigma}_f\right)& 0\le N\left(z,{\upsigma}_f\right)\le 100\\ {}100& N\left(z,{\sigma}_f\right)>100\end{array}\right. $$

where, in Eq. 4a, *N*(*μ*, *σ*) is the Gaussian noise we added to *q* values (vectorized to the size of number of variants, each taking a Gaussian random number with mean of *q*_1_ and *q*_2_, normalized such that $\frac{1}{L}\left(N\left({q}_1,{\sigma}_{q_1}\right)+N\left({q}_2,{\sigma}_{q_2}\right)\right)=1$ . It followed by another Gaussian noise σ_*f*_ added to the FREQ, which we will change from 0 to 20.

## Results

Cell line misidentification and contamination is a common problem affecting the reproducibility of cell-based research and therefore cell line authentication becomes really important. SNV profiles have been used earlier to re-identify the lung and colorectal cancer cell lines as well as HeLa contamination but these studies were limited to only few cell lines [5, 11]. In this study we have made an attempt to use variants derived from RNA-seq data for large-scale cell line authentication.

### Variant analysis

RNA-seq data for 934 cell lines available from the NCI GDC legacy portal (https://portal.gdc.cancer.gov/) were downloaded and bam files were processed to call variants using an in-house pipeline described earlier in the methods section. Additionally, WES data for 326 cell lines available from GDC were also obtained and variants were identified. A total of 1,027,428 of variants were identified across all the cell lines with an average of 27,310 variants per cell line. As shown in Fig. 1a, all variant profiles of RNA-seq samples will be used to determine their correlation coefficient distribution and its corresponding significance level from CCLE collection, and the process to determine the CeL-ID accuracy and its robustness, followed by a validation procedure utilizing a collection of independently obtained MCF7 and HCT116 cells processed with different treatment [24, 25], and down-sampling of RNA-seq samples to explore how little sequence reads are required to achieve the equivalent identification accuracy.

### Cell line authentication using variant comparisons

**Fig. 2:**
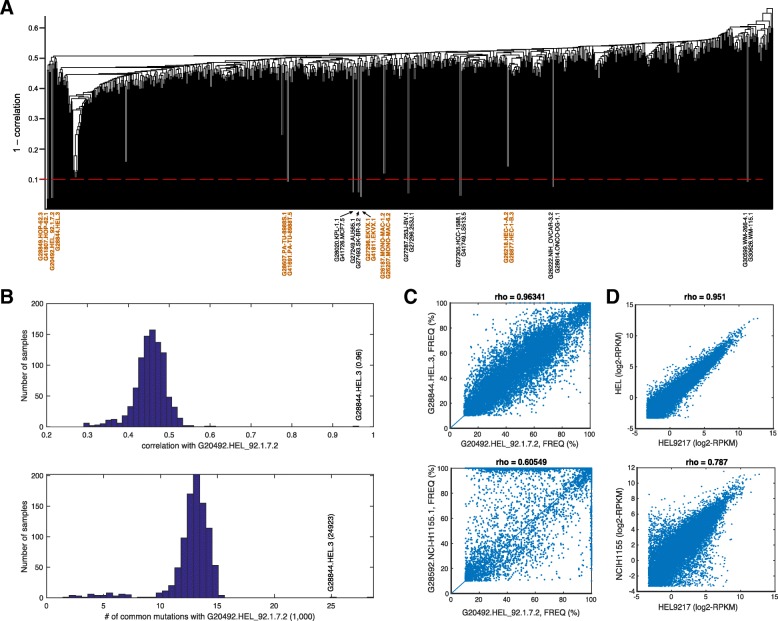
Correlation coefficient and hierarchical clustering. (**a**) Pairwise correlation coefficients for all 934 cell lines were calculated and cell lines pairs with highest correlations are listed on x-axis (samples shown in brown color are replicate or identical pairs used in Fig. 3b); (**b**) shown are the correlation coefficient and number of common mutations between sample G20492.HEL_92.1.7.2 and others. The best matched sample G28844.HEL.3 is marked on both plots; and (**c**) & (**d**) scatter plots of G20492.HEL_92.1.7.2 with its best match (top) and second best-match (bottom) using variant (**c**) frequencies (%) and (**d**) gene expression (rpkm) values

We performed the pair-wise comparisons of variant profiles of all the 934 cell lines and computed correlation coefficients. It is interesting to note that only a few pairs of cell lines showed high correlation coefficients (*ρ* > 0.8) whereas most other pairs show poor correlation (Fig. 2a and b). Moreover, most of the top identified cell line pairs with correlations (*ρ* > 0.9) were turned out to be known replicates, subclones, derived from same patients or have been known in the literature to share high SNP identity (CCLE legacy archive (https://portals.broadinstitute.org/ccle/data); Fig. 2a and b). As can be seen in Fig. 2a, correlation coefficients were used as distance metric to carry out hierarchical clustering. CCLE dataset happened to include replicates for two cell lines sequenced at different time and our CeL-ID method correctly identified these two pairs: G28849.HOP-62.3 & G41807.HOP-62.1 (*ρ* = 0.97), and G27298.EKVX.1 & G41811.EKVX.1 (*ρ* = 0.96). Moreover, pair – G20492.HEL_92.1.7.2 & G28844.HEL.3 also identified to be very similar (*ρ* = 0.96; Fig. 2c) are known to be subclones, whereas cell line pairs: G27249.AU565.1 & G27493.SK-BR-3.2, G30599.WM-266-4.1 & G30626.WM-115.1 and G28607.PA-TU-8988S.1 & G41691.PA-TU-8988 T.5 (cell line names are shown in Fig. 2a) were known to be derived from the same patient and hence share high variant identity. Additionally, other four pairs including the cell line pair G41726.MCF7.5 & G28020.KPL-1.1 were known to share high SNP identity and in some cases literature indicates that they are same or likely to be the same, for example, G27305.HCC-1588.1 is likely to be G41749.LS513.5 and G28614.ONCO-DG-1.1 is likely to be G26222.NIH_OVCAR3.2 (https://portals.broadinstitute.org/ccle/data). Majority of cell line pairs rightly show poor correlation (*ρ* < 0.6, Fig. 2a and b). The only anomaly we observed is from a subset of six cell lines (G27483.S-117.2, G28592.NCI-H155.1, G28551.MHH-CALL-2.1, G28045.KYSE-270.1, G27239.ACC-MESO-1.1 and G28088.LOU-NH91.1), which show pretty high correlation with each other (*ρ* = 0.83–0.89) but have different cells of origin and derived from different cancers. These cell lines may just happen to share high variant identity or somewhere during the cell culturing and maintenance cells got contaminated with each other. As expected, correlated cell lines tend to share more common mutations (Fig. 2b).

Transcriptome profiles of any given cells are known to change during various treatments, and adapt to their environment as well. For base-line expression data provide through CCLE project, we can see their correlation holds for pair G20492.HEL_92.1.7.2 & G28844.HEL.3 (*ρ* = 0.95, Fig. 2d), and the next-to-best correlated sample is also NCI-H1155 (*ρ* = 0.787). Notice the difference of correlation coefficients of the best sample and the next-to-best samples are much smaller than those derived from variant profiles.

Furthermore, we analyzed WES data for 326 cell lines available from NCI GDC. These 326 cell lines include 112 cell lines from the RNA-seq dataset. All the variants from WES data were identified using pipeline showed in Fig. 1a. We used variants derived from WES data to compare it with those of RNA-seq and a high degree of concordance was observed.

### Determination of the significance of correlation coefficient

**Fig. 3:**
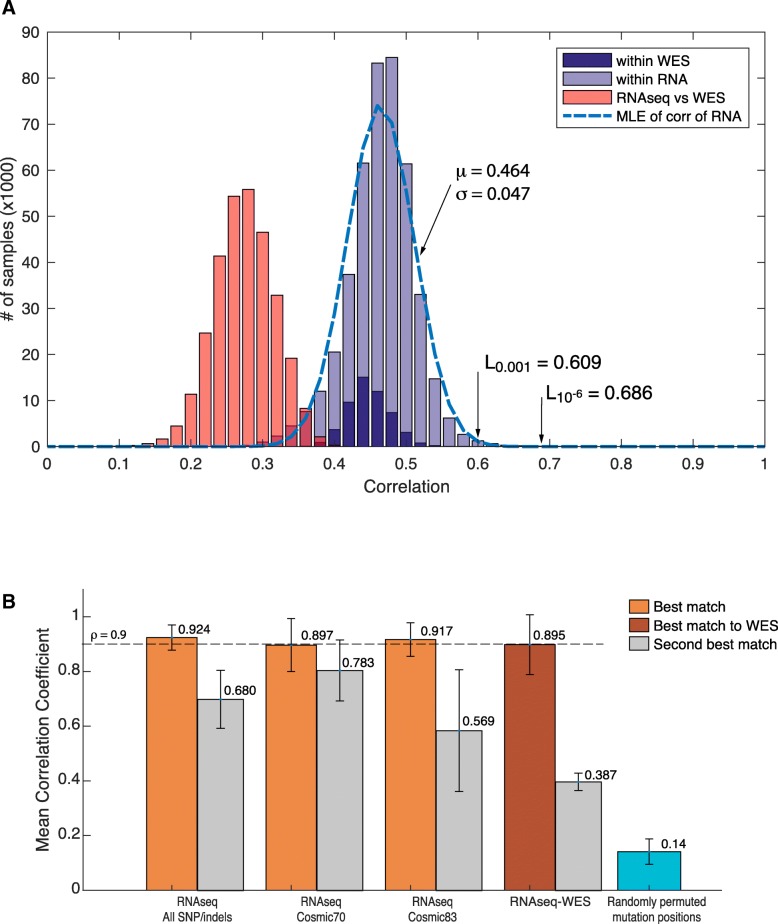
Distribution plot and test accuracy. **a** Shown are distribution plots of pairwise correlation coefficients in 934 RNA-seq (light blue), 326 WES datasets (dark blue), and correlations between RNA-seq and WES data. The estimated normal distribution is also plotted in black line; and (**b**) Mean correlation coefficients (of 6 replicate pairs highlighted in brown color in Fig. 2a) obtained for the best match and the second best match using all variants, COSMIC70 and COSMIC83 constrained variants, RNAseq-WES variants and randomly permuted mutation positions

Moreover, to determine the significance of a detected correlation coefficient for a given cell line, all pair-wise correlations for 934 cell lines were generated. Distribution plot of correlation follows normal distribution N(μ,σ) (Fig. 3a, light blue histogram). Similar distribution is also observed in pair-wise correlation from WES samples (Fig. 3a, dark blue histogram). To estimate parameter distribution, we used truncated normal distribution model by removing correlation coefficients less than 0 (unlikely) and greater than 0.8 (replicate and derivative cell-lines in CCLE collection). For variant profiles derived from RNA-seq, parameters are (*μ*, *σ*) = (0.464, 0.047). Therefore, at *L*_0.001_ = 0.609, two samples will be considered similar with *p* < 0.001, or at *L*_10_^-6^ = 0.686 two samples will be unlikely similar (*p* < 10^− 6^). As a comparison, between RNA-seq and WES variant profiles (*μ*, *σ*) = (0.275, 0.042), excluding all pair-wise comparison between same cell lines (see Fig. 3a, left pink histogram).

### COSMIC SNVs and cell line re-identification

We constrained the variants being used for correlation calculation to only those present in COSMIC70 and COSMIC83 databases [18]. This led to a huge reduction in number of variants. Only 4% of total variants matched to COSMIC70 and 14% matched to the latest cosmic database COSMIC83 (Table 1). To test the validity of using only the cancer mutations, we selected 6 pairs of cell lines that have either replicate or derivative cell lines in CCLE dataset (G41807.HOP-62.1, G28844.HEL.3, G27298.EKVX.1, G28607.PA-TU-8988S.1, G26187.MONO-MAC-1.2 and G26218.HEC-1-A.2, highlighted in brown color in Fig. 2a). Interestingly, we observed that only COSMIC matched variants are sufficient to correctly re-identify the cell lines (Fig. 3b). Only COSMIC70 showed relative poor performance with 2nd best match (beyond the pair) due to its lower number of SNPs for comparison. We note that using COSMIC mutation takes much less computation time for correlation coefficient evaluations across all cell lines.

**Table 1:** Total number of variants: Number and percentage of variants matched to COSMIC70 and COSMIC83 are given. A total of 1,027,428 variants were detected across all the cell lines

COSMIC Dataset	Number of variants matched	Percentage of matched variants (%)
COSMIC70	40,742	3.96
COSMIC83	143,923	13.91

### Robustness of the model

**Fig. 4:**
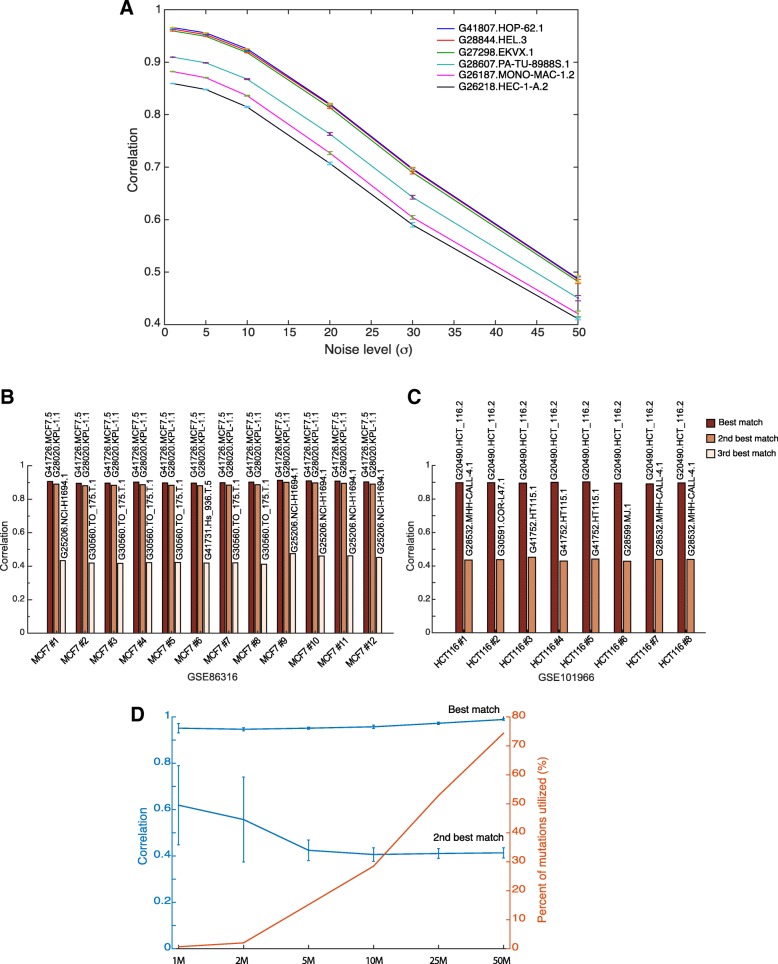
Test for the robustness of the model. **a** Shown are effect of adding noise to data with 6 pairs used in Fig. 3b; **b** test on an independent set of 12 MCF7 RNA-seq datasets of GSE86316, with their first best-match MCF7, second best-match KLP-1 and the third candidate (not consistent for all 12 MCF7 samples); **c** test on an another independent set of 8 HCT116 RNA-seq datasets of GSE101966; and (**d**) effect of sequencing depth on prediction accuracy using subsampling (1 M, 2 M, 5 M, 10 M, 25 M and 50 M) method

We tested the robustness of CeL-ID method by adding noise (Gaussian noise with zero mean) to the allele frequency of variant data for six pairs of cell lines as aforementioned. As evident from the Fig. 4a, correlation drops significantly with increasing noise level and by the noise level *σ* = 15~20 cell line pair is not identifiable. Additionally, to estimate the false positive rate, we randomly permuted the mutation positions in these six cell lines and tried to find the other pair. We repeated it 100 times and as can be seen in Fig. 3b (last bar), with very low correlation coefficient (on average, *ρ* = 0.14).

Moreover, we tested the robustness of CeL-ID method on two independent test sets. First independent test set comprises of 12 RNA-seq datasets for MCF7 cells, which were downloaded from GEO (GSE86316) and represents mRNA expression profiles in MCF7 cells after silencing of MEN1 using small hairpin or chemical inhibition that affected expression profile of selected group of transcripts [24]. The second independent set consists of 8 RNA-seq datasets for HCT116 cells. These were also obtained from GEO (GSE101966) and depict mRNA expression profiles in HCT116 cells after loss of ARID1A and ARID1B [25]. Variants were called using pipeline (Fig. 1a, light blue boxes) and as can be seen in Fig. 4b and c, even variants derived from altered mRNA expression profiles are sufficient for authentication/re-identification of cell lines. Additionally, it may be noted that even the use of a different aligner RSEM do not affect cell re-identification potential. As mentioned earlier, MCF-7 and KPL-1 are known to share high SNP identity and hence both rightly passed threshold for unique identification. We removed variants that shared between these two cell lines with difference FREQ greater than 10 and high coverage depth requirement, reducing 17,730 variants in first pass to 2631. Detail analysis results are provided in Table 2. Notice that second pass *p*-value is much higher, which is due to the removal of common variants, only assess the agreement with variant sites perhaps differentiate MCF7 and KPL-1. Similar results were also obtained for HCT116 cells and are provided in Additional file 2.

**Table 2:** Test results on independent test set of 12 MCF7 cells obtained from GSE86316. Two passes of the cell identification process was invoked since two cell lines (G41726.MCF7.5 and G28020.KPL-1.1) both pass the correlation/*p*-value test. By removing common variants between two cells and with deep coverage requirement, the difference of correlations is much larger than the first test

Samples	First test with all variants (17,730)			Second test after removing common variants (2631)		
	Match cell names	corr. Coef	p-value	Match cell names	corr. Coef	p-value
Sample 1	G41726.MCF7.5	0.91	8.59E-21	G41726.MCF7.5	0.53	0.084
	G28020.KPL-1.1	0.89	1.74E-19	G28020.KPL-1.1	0.34	0.996
	G25206.NCI-H1694.1	0.43	0.781			
sample 2	G41726.MCF7.5	0.9	6.63E-20	G41726.MCF7.5	0.49	0.309
	G28020.KPL-1.1	0.88	1.07E-18	G28020.KPL-1.1	0.32	1
	G30560.TO_175.T.1	0.42	0.863			
sample 3	G41726.MCF7.5	0.9	5.14E-20	G41726.MCF7.5	0.5	0.266
	G28020.KPL-1.1	0.88	6.73E-19	G28020.KPL-1.1	0.35	0.993
	G30560.TO_175.T.1	0.42	0.869			
Sample 4	G41726.MCF7.5	0.9	1.64E-20	G41726.MCF7.5	0.55	0.053
	G28020.KPL-1.1	0.89	3.50E-19	G28020.KPL-1.1	0.37	0.983
	G30560.TO_175.T.1	0.42	0.847			
sample 5	G41726.MCF7.5	0.9	4.22E-20	G41726.MCF7.5	0.49	0.304
	G28020.KPL-1.1	0.88	7.66E-19	G28020.KPL-1.1	0.32	0.999
	G30560.TO_175.T.1	0.42	0.843			
sample 6	G41726.MCF7.5	0.9	5.94E-20	G41726.MCF7.5	0.5	0.283
	G28020.KPL-1.1	0.88	1.10E-18	G28020.KPL-1.1	0.31	1
	G41731.Hs_936.T.5	0.42	0.859			
sample 7	G41726.MCF7.5	0.9	3.48E-20	G41726.MCF7.5	0.51	0.185
	G28020.KPL-1.1	0.88	6.27E-19	G28020.KPL-1.1	0.35	0.995
	G30560.TO_175.T.1	0.42	0.854			
sample 8	G41726.MCF7.5	0.9	1.90E-20	G41726.MCF7.5	0.52	0.122
	G28020.KPL-1.1	0.89	3.88E-19	G28020.KPL-1.1	0.33	0.999
	G30560.TO_175.T.1	0.41	0.89			
sample 9	G41726.MCF7.5	0.91	1.89E-21	G41726.MCF7.5	0.56	0.025
	G28020.KPL-1.1	0.9	2.53E-20	G28020.KPL-1.1	0.39	0.954
	G25206.NCI-H1694.1	0.48	0.455			
sample 10	G41726.MCF7.5	0.91	3.85E-21	G41726.MCF7.5	0.55	0.037
	G28020.KPL-1.1	0.9	4.70E-20	G28020.KPL-1.1	0.38	0.971
	G25206.NCI-H1694.1	0.46	0.572			
sample 11	G41726.MCF7.5	0.91	5.55E-21	G41726.MCF7.5	0.54	0.058
	G28020.KPL-1.1	0.89	7.66E-20	G28020.KPL-1.1	0.38	0.966
	G25206.NCI-H1694.1	0.46	0.571			
sample 12	G41726.MCF7.5	0.9	1.42E-20	G41726.MCF7.5	0.53	0.086
	G28020.KPL-1.1	0.89	1.68E-19	G28020.KPL-1.1	0.37	0.985
	G25206.NCI-H1694.1	0.45	0.642			

Note:

1. First test takes all variants with DP > = 10, and at least one sample FREQ > 0. Total of 17,730 variants are included

2. Second test takes variants with DP > = 20, and the difference of max(FREQ of MCF7 and KPL-1.1) and min(FREQ of MCF7 and KPL-1.1) > 10. Total of 2631 variants are taken for all 12 samples’ second test

Furthermore, to test the robustness of the system, effect of sequencing depth on the results was checked. We randomly selected nine cell lines and randomly subsampled it to 1 million (1 M), 2 million (2 M), 5 million (5 M), 10 million (10 M), 25 million (25 M), and 50 million (50 M) reads and ran the pipeline on subsampled subset of reads. As evident from the Fig. 4d, even smaller subset of up to 5 M reads covering only around 15% of total variants (red line/right axis, Fig. 4d) are enough for cell line authentication (top blue line/left axis, Fig. 4d). Similar results were observed for all subsampled sets from all nine cell lines, as indicated by small error bars (Fig. 4d), demonstrating that our method is robust enough up to 5 M reads sequencing depths. Only notable observation is the variation of correlation for the second best-match (lower blue line/left axis, Fig. 4d) increases with the reduction of total read counts, particularly at 1 M and 2 M read count levels, indicating lower read counts will render much fewer unique variants available for mutation calling, and increases the chance of false positive.

### Sample mix-up and contamination estimation

**Fig. 5:**
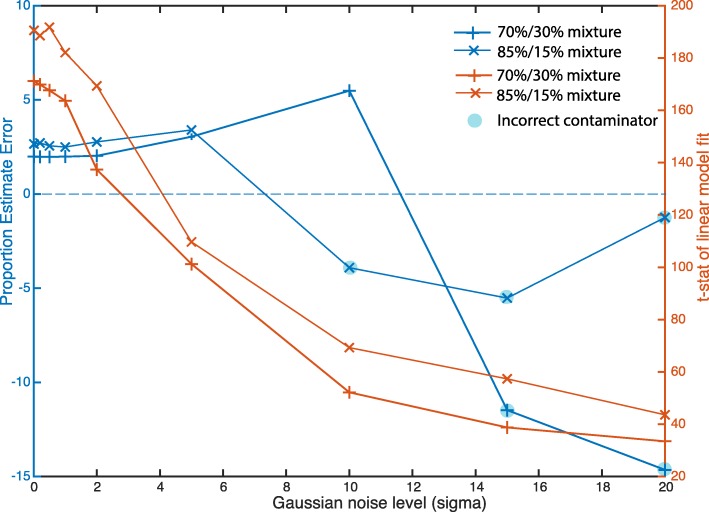
Contaminant estimation using linear mixture model. Shown are plots of contaminant estimate and t-stat for linear model fit for varying Gaussian noise level

Cell line contamination is a major issue facing biomedical sciences [1, 9]. Human error and oversight are thought to be the main cause of cell line mix-ups and contamination. It’s necessary to have means to quality control these errors rapidly and periodically. Henceforth, we have developed a linear regression model (see Methods section, Fig. 1b) to estimate the level of mix-ups and contamination using variant frequencies from RNA-seq data. To evaluate the effectiveness of the deconvolution method, we first simulated observed data by mixing two selected frequencies datasets using Eqs. 4a and 4b. The exact steps are provided below:

1. For example, we select G20492.HEL_92.1.7.2 as test sample (*x*_1_), and G20469.JHOS-2.2 as contaminant candidate (*x*_2_);
2. Generate proportion *q*_1_ from a normal distribution with mean 0.85 and standard deviation 0.05 (or *q*_1_ ~ *N*(0.85, 0.05), and *q*_2_ ~ *N*(0.15, 0.05). We also tested proportion of 0.70/0.30, as shown in Fig. 5;
3. Following Eq. 4a, we have *z* = *q*_1_ ∙ *x*_1_ + *q*_2_ ∙ *x*_2_ for both FREQ and DP;
4. For each standard deviation *σ*_*noise*_ = (0.01, 0.1, 0.2, 0.5, 1, 2, 5, 10, 15, 20), we perform,
  4.1. Following Eq. 4b, we obtained *y* = *z* + *N*(0, *σ*_*noise*_) only for FREQ, and then reset y to 0 if y < 0, and 100 if y > 100;
  4.2. Calling function CCLE_Identification() to identify dominant cell-line, or provide cell line identification. For our particular example selection and for *σ* = 0.01, we obtained:
    1st match cell-line = G20492.HEL_92.1.7.2, with *ρ* = 0.97, *p* = 9.762 × 10^− 27^
    2nd match cell-line = G28844.HEL.3, with *ρ* = 0.90, *p* = 1.768 × 10^− 20^
    3rd match cell-line = G25242.K-562.3, with *ρ* = 0.47, *p* = 0.514

Note that 2nd matched cell line (HEL.3) is the cell line that has a high correlation with HEL_92.1.7.2 (See Fig. 2a), and we expect it to be identified before the 3rd best match;

4.3. Calling function CCLE_MixtureEstimate() to identify contaminant and *q*_1_ and *q*_2_. Results for our demonstrated case with (*σ* = 0.01, and 85/15 mixture);

- The possible mixture is G20469.JHOS-2.2, with proportion q2 = 82.3%, with t-stat = 210.0, p-value 0.000000e+00

The identified cell line is the same as we started with, and proportion is 82.3% (or − 0.27 below the targeted 0.85 level); and

5. Report estimate results in Fig. 5.

As evident from Fig. 5, that the linear model regression method can correctly estimate the level of contaminator to an extent. The linear model tends to slightly under-estimate the proportion (about 3%, for both 70%/30 and 85%/15% mixtures, blue line, Fig. 5) for simulated noise *σ* from 0 to 6. With the increase of the s, the t stats for each proportion variable estimate decreases (2 red lines, Fig. 5), at some noise level, the proportion will over-estimate the correct level (blue lines cross zero, Fig. 5), which indicates the inability of the linear model regression to identify a correct contaminator from 934 cell-line collections (indicated by a blue circle, Fig. 5). The best case scenario would have been to show the estimation accuracy on a real mixed test dataset and we will continue to investigate the availability of such dataset.

## Discussion

In this study we describe a method (CeL-ID) for estimating cell line purity from RNA-seq data. A key advantage of using the CeL-ID method for cell line authentication is that it relies on a complete set of variants from the transcriptome instead of a fixed panel of small numbers of STRs or SNPs, and hence avoids the loss of statistical power caused by allelic dropout that affects STR-based authentication methods [1, 9–11]. This becomes more pressing in case of cancer cell lines where genetic instability is prevalent and known to exhibit aneuploidy and microsatellite instability [2, 3, 11].

Currently, STR profiling is the ANSI standard for authenticating cell lines [2]. STR profiles for a large number of cell lines are available for comparison, and a growing number of fee-for-service companies provide STR-based cell line authentication for a cost ranging from $100–295 [9, 10]. SNP-based profiling methods had been developed as a simple and stable alternative but suffer from lack of accessibility and being too cumbersome for many researchers. Whereas CeL-ID was developed on the premise that a significant number of cell-based studies anyway employs RNA-seq-based transcriptome profiling in their research and the same can also be used to ascertain the identity of the cell line. In this way, researchers will save both the money and effort of separately authenticating the cell line.

Benchmarking studies on independent test sets showed that CeL-ID method is precise and robust and can be used as a resource for cell line authentication. Genentech authenticated cell lines contain a consolidated list of 3587 cell lines [1], of which we had access to RNA-seq data for more than 900 cell lines covering most of the commonly used cell lines. We have generated and stored variant profiles for these 900 plus cell lines for comparison and will keep updating the database as we have access to RNA-seq data for additional cell lines. Therefore, as an end-user one just has to input either an alignment (bam) file or variant (vcf) file for a given cell line and CeL-ID will carry out all the pairwise comparisons and output the perfect match and will also estimate about the possible contaminants if no perfect match was detected.

## Conclusions

In summary, we have developed a new method called CeL-ID, for cell line authentication using variant profiles derived from RNA-seq data and has shown its robustness. CeL-ID successfully identifies identical, synonymous and derivative cell lines and also estimates about the possible contaminant. We have attempted to provide simple solution to problem associated with cell line authentication and hope this would help in adoption of regular cell line authentication.

## Additional files

Additional file 1: List of sample names in CCLE dataset (BG-04-S1.xls). (XLS 92 kb)

Additional file 2: Test results on an independent test set of eight HCT116 cells obtained from GSE101966 (BG-04-S2.xls). (XLS 38 kb)

## Abbreviations

AD: Allele Depth
ATCC: American Type Culture Collection
CCLE: The Cancer Cell Line Encyclopedia
CeL-ID: Cell Line Identification
DP: Depth of Coverage
FREQ: Frequency
GDC: Genomic Data Commons
NCI: National Cancer Institute
STR: Short Tandem Repeat
WES: Whole Exome Sequencing
WGS: Whole Genome Sequencing
